# Exposure Science and the Exposome: An Opportunity for Coherence in the Environmental Health Sciences

**DOI:** 10.1289/ehp.1104387

**Published:** 2011-11-01

**Authors:** Paul J. Lioy, Stephen M. Rappaport

**Affiliations:** Environmental and Occupational Health Sciences Institute, Robert Wood Johnson Medical School–UMDNJ and Rutgers University, Piscataway, New Jersey; School of Public Health, University of California, Berkeley, California, E-mail: srappaport@berkeley.edu

The field of exposure science began with qualitative observations and quantitative measurements of air contaminants to aid our understanding of exposure–disease relationships. In fact, some of the earliest writings that describe the essence of exposure science are found in Bernardino Ramazzini’s 1700 treatise on occupational diseases (Franco 1999). In the 1920s, exposure scientists collaborated with epidemiologists to investigate workplace exposures as sources of occupational diseases (Rappaport 2011). Between the 1950s and 1970, investigations expanded to include exposures to pollutants in ambient and indoor air and water (Rappaport 2011). Following establishment of U.S. governmental agencies in the 1970s to regulate exposures in the workplace (Occupational Safety and Health Administration) and the ambient environment [U.S. Environmental Protection Agency (EPA)], the paths of exposure scientists diverged into those investigating sources of pollutants in occupational settings and those investigating ambient sources of pollutants (Rappaport 2011). By the 1990s the two groups had essentially parted ways, and the term “exposure science” was associated with community and personal exposures to ambient pollutants (Lioy 2010; Ott 1990, 1995). Investigations of total personal exposure initially employed external measurements of chemicals that can enter the body by inhalation, ingestion, and dermal contact (1970s), and internal markers of exposure were added in the 1980s and 1990s (Centers for Disease Control and Prevention 2009; Hoffmann et al. 2000; Sexton et al. 1995; Wallace et al. 1985). In the 21st century, exposure science has increasingly embraced deterministic models to predict levels of diverse exposures based on categorical data (Cohen Hubal et al. 2010; Georgopoulos and Lioy 2006; Lioy 2010) and on measured levels of pollutants in biological fluids and tissues (Georgopoulos et al. 2009).

In parallel with the above activities, during the 1980s and 1990s, molecular epidemiologists explored links between genetic and environmental factors and the resulting biochemical or biological indicators of possible ill health (biomarkers) measured in individual subjects (Bonassi and Au 2002). When completion of the human genome project in 2000 made it feasible to measure thousands of polymorphic genes in each subject, epidemiology increasingly focused on the genetic determinants of diseases (Hindorff et al. 2009). However, as results of these genome-wide association studies (GWAS) failed to explain most variability in human diseases (Manolio et al. 2009), interest in environmental factors reemerged. But there was no environmental analog of GWAS; that is, we had no way of characterizing the totality of a person’s environmental exposures. This prompted Christopher Wild to publish a commentary that defined the “exposome” as the environmental complement to the genome (Wild 2005). Recognizing that humans are exposed to health-impairing agents from both pollution and nonpollution sources and that these sources change during a lifetime, Wild indicated that “… the exposome encompasses life-course environmental exposures (including lifestyle factors) from the prenatal period onwards.” This is a powerful idea because it considers a person’s lifetime history of all exposures experienced from both external sources (e.g. pollution, radiation, and diet) and internal sources (e.g. inflammation, infection, and the microbiome) (Rappaport and Smith 2010). Thus, one can imagine a future in which individuals’ exposomes are contrasted between diseased and healthy populations for molecular epidemiology, or over different life stages as part of personalized medicine (Nicholson 2006). In either case, the goal would be to discover causes of ill health and to generate hypotheses regarding identification and elimination or reduction of harmful exposures.

If the exposome concept is to be useful to exposure science, methods will be needed to characterize individual exposomes and to investigate sources of exposome variability. Because exposures arise from diverse sources, Rappaport defined two generic approaches for characterizing exposomes (Rappaport 2011; Rappaport and Smith 2010). A “bottom-up” approach would focus on each category of external exposure—including air, water, diet, radiation, lifestyle, etc.—to quantify contaminant levels that would be summed over all categories to estimate individual exposomes. This approach is appealing to some exposure scientists because it focuses on the same external media that have long been investigated and leads logically to interventions for eliminating or reducing exposures. However, this bottom-up approach would require tremendous effort to evaluate the myriad of largely unknown analytes in various external media and would also miss important endogenous exposures. The alternative “top-down” approach would adopt untargeted omic methods to measure features of exposures in biological fluids, and thus finds appeal with exposure scientists who have used biomonitoring for assessing exposure levels, albeit on a chemical-by-chemical basis. This approach is more efficient because both exogenous and endogenous exposures would be represented by a single specimen of blood, for example, and would encourage contrasts of omic profiles between diseased and healthy populations in much the same manner as GWAS (Patel et al. 2010). Omic profiles would generate hypotheses to *a*) indentify particular exposures, *b*) develop specific biomarkers for high-throughput screens, and *c*) determine sources of external and internal exposure. Recent untargeted metabolomic studies have applied this top-down approach to identify hitherto unknown exposures associated with cardiovascular disease (Holmes et al. 2008; Wang et al. 2011).

When examined objectively, there is scientific value in both the bottom-up and top-down approaches for characterizing individual exposomes. The top-down approach offers appeal for discovering unknown causes of human disease (Rappaport 2011; Rappaport and Smith 2010), whereas the bottom-up approach encourages more comprehensive analyses of external exposures and methods for intervention and prevention (Lioy 2010). Indeed, we envision long-term strategies that embrace elements of both approaches for improving public health. Unfortunately, the differentiation between external (air, water, soil/dust, etc.) and internal (biological fluids) media has led to an apparent disconnect or competition between exposure scientists who focus on external monitoring and modeling and those who favor biomonitoring and omic methods. Indeed, we are encountering a view that can be summarized as “exposure science versus the exposome.” This is counterproductive because it potentially deprives exposure science of avenues for vastly diversifying its pool of relevant exposures and for strengthening the source-to-dose framework needed by the environmental health sciences. Rather than adopting defensive postures, we encourage exposure scientists to exploit the relative strengths of both monitoring approaches for assessing human exposures. Toward this end, the National Academy of Sciences will convene a workshop in December 2011 to better integrate the top-down and bottom-up approaches for characterizing individual exposomes (Emerging Technologies for Measuring Individual Exposomes, 8–9 December 2011, Washington, DC; information is available at http://dels.nas.edu/envirohealth).

Other recent developments offer opportunities for exposure scientists to characterize individual exposomes. For example, the National Children’s Study (Landrigan et al. 2006) offers an evolving platform with which to link the top-down and bottom-up approaches. Because individual data and biospecimens will be collected during the first 21 years of life, this study will provide resources that can be used to evaluate the variability of exposome features during critical life stages. Moreover, the extensive questionnaire data, home samples, extant environmental data, and dietary histories of participants suggest avenues for modeling connections between the internal and external environments. Such prospective cohort studies will allow us to collect more and better exposure data with which to identify unknown health hazards and to develop appropriate preventive measures and regulations for recognized hazards. The exposome concept can play a key role in both endeavors.
